# Population-based incidence, mortality and quality of life in critically ill patients treated with renal replacement therapy: a nationwide retrospective cohort study in finnish intensive care units

**DOI:** 10.1186/cc11158

**Published:** 2012-01-20

**Authors:** Suvi T Vaara, Ville Pettilä, Matti Reinikainen, Kirsi-Maija Kaukonen

**Affiliations:** 1 Intensive Care Units, Division of Anaesthesia and Intensive Care Medicine, Department of Surgery, Helsinki University Central Hospital, Box 340, 00029 HUS, Finland; 2 Department of Intensive Care, North Karelia Central Hospital, Tikkamäentie 16, 80210, Joensuu, Finland

## Abstract

**Introduction:**

Acute kidney injury (AKI) increases mortality and morbidity of critically ill patients. Mortality of patients treated with renal replacement therapy (RRT) is high. We aimed to evaluate the nationwide incidence of RRT-treated AKI in Finland, hospital and six-month mortality, and health-related quality of life (HRQoL) of these patients.

**Methods:**

We performed a retrospective cohort study including all general intensive care unit (ICU) admissions in Finland in 2007 through 2008. We identified patients who had received RRT due to AKI (RRT patients) and compared these patients to ICU patients who were not treated with RRT (non-RRT patients). The HRQoL was assessed by the EQ-5D index and visual analogue scale (VAS).

**Results:**

We analysed the final cohort of 24,904 patients, of whom 1,686 received RRT due to AKI. The incidence of RRT-treated AKI was 6.8% (95% confidence interval (CI) 6.5 to 7.1%) among ≥ 15-year-old general ICU patients, which corresponds to a yearly population-based incidence of 19.2 per 100,000 (95% CI 17.9 to 20.5/100,000). According to RIFLE (Risk, Injury, Failure) classification 26.6% (95% CI 26.0 to 27.2%) of patients had AKI (RIFLE R-F). Hospital and six-month mortality of RRT patients were 35.0% and 49.4%. At six-months, RRT patients perceived their health as good as non-RRT patients by VAS.

**Conclusions:**

The population-based incidence of AKI treated with RRT was 19.2 per 100,000 in Finland and 6.8% of all general ICU patients. The hospital and six-month mortality rates were lower than previously reported for ICU-treated RRT patients.

## Introduction

Acute kidney injury (AKI) increases mortality, length of stay (LOS) and resource need in intensive care unit (ICU) patients. The incidence of AKI has been reported to vary between 6 and 70% among ICU patients depending on the definition [1-3]. The RIFLE (Risk, Injury, Failure) -classification for AKI based on serum creatinine concentration and urine output was published in 2004 [4]. Patients receiving renal replacement therapy (RRT) represent the most severe form of AKI.

The incidence of RRT-treated AKI in populations has been reported to vary between 8 and 30 per 100,000/year [5-12] with an increasing trend [11,12]. The incidence of RRT-treated AKI among general ICU patients lies between 4 and 8% [1,3,13,14]. Hospital mortality of these patients has been reported to be 44 to 64% [5,13,15-19]. Higher disease severity [3], use of vasoactive drugs, mechanical ventilation, sepsis [1] and longer hospital stay prior to ICU ad-mission [17] are associated with increased hospital mortality. Surgical admission is related with better outcome [17,19]. Furthermore, health-related quality of life (HRQoL) of RRT-treated AKI patients has been reported to be lower than in general population [20]. However, the number of patients in these studies has been limited [6,20,21]. Studies presenting the incidence of RRT-treated AKI in general ICU patients along with long-term outcome and HRQoL are scarce.

Accordingly, we aimed to evaluate the nationwide incidence of RRT-treated AKI in Finland, and their hos-pital and six-month mortality, and their HRQoL at baseline and after six months follow-up.

## Materials and methods

The board of the Finnish Intensive Care Consortium (FICC) approved the study protocol. The Ethics Committee of the Department of Surgery, Hospital District of Helsinki and Uusimaa waived the need for an informed consent. The consortium maintains a database, where detailed data on patient characteristics, disease severity scores, and patient outcomes are prospectively collected and validated. During the study period from 1 January 2007 to 31 December 2008, the database included all admissions in nine ICUs of five Finnish University hospitals and in 15 central hospitals and included 30,380 ICU admissions. Four highly specialised units were not members of the consortium, but the number of RRT-treatments in these units is minimal. Thus, all general ICUs providing RRT were included in the study.

We searched the database for patients who had received RRT due to AKI. RRT and the day of RRT initia-tion were registered in the database. For comparison, we obtained the same data on all ICU patients for the same period. For all analyses we excluded readmissions, patients under the age of 15, patients with end-stage renal disease (ESRD) requiring dialysis prior to ICU admission based on chronic health evaluation, and patients admitted because of drug or alcohol intoxi-cation.

The data of each patient treated in the ICU during the study period included 1) demographic data, 2) SAPS II (Simplified Acute Physiology Score) [22] and SOFA (Sequential Organ Failure Assessment) severity scores and organ spe-cific SOFA scores [23], 3) intensity of care measured by TISS (Therapeutic Intervention Scoring System) score [24], 4) APACHE (Acute Physiology and Chronic Health Evaluation) III and ICD-10 (International Classification of Diseases, 10^th ^revision) diagnosis and the APACHE III diagnosis group, 5) physiological data, and 6) laboratory values. In addition to daily creatinine values, we obtained the data of the severity scores as well as physiological and laboratory values from the first 24-hour ICU-treatment period. If data were missing for more than 5% of cases, we indicate it in the tables. We used the Finnish population data from Statistics Finland for the epidemiological calculations. On 31 December 2007, Finland (except the Åland Islands) had 4,383,358 ≥ 15-year-old inhabitants. We analysed the data on ICU and hospital length of stay, hospital mortality and six-month mortality. We calculated the standardised mortality ratio (SMR, the number of observed hospital deaths divided by the number of expected deaths) according to the original SAPS II equation [22].

We classified the patients according to the maximum RIFLE class [4] during their ICU stay. Due to missing urine output data we only used the glomerular filtration rate (GFR) criteria: RIFLE-Risk serum creatinine ≥ baseline creatinine × 1.5; RIFLE-Injury serum creatinine ≥ baseline × 2; RIFLE-Failure serum creatinine ≥ baseline × 3 or > 354 micromol/l with an acute rise > 44 micromol/l [4]. As baseline creatinine we used the lowest value of the follow-ing: the lowest creatinine during the patient's ICU stay (in 76% of the cases) or calculated creatinine from the MDRD (Mod-ification in Diet in Renal Disease) equation [25] assuming a glomerular filtration rate of 75 ml/minute/1.73 m^2 ^as recom-mended by ADQI (Acute Dialysis Quality Initiative) [4]. We determined the day of RRT initiation and the preceding RIFLE class.

We analysed the HRQoL with the EuroQol (EQ-5D) instrument [26,27]. EQ-5D includes five dimensions: mobility, self-care, usual activities, pain/discomfort and anxiety/depression. Each dimension is scored from 1 to 3 and population-based preference weights are used to calculate the index score (maximum value 1) (Additional file 1). The instrument also includes a visual analogue scale (VAS) ranging from 0 to 100 (100 represents the best) for self-rating the health state. The baseline values were obtained during the patients ICU stay by interviewing either the patient himself or his proxy. For follow-up, the query was performed by a phone interview or a letter, depending on the practice in each centre. We calculated the EQ-5D index score and analysed it and the VAS score at baseline and after six months follow-up among patients who were reported to be alive at six months.

To compare septic and non-septic patients, we searched the patients with infection diagnosis by screening the APACHE III and ICD-10 diagnoses. We screened all patients for fulfilling the SIRS (systemic inflammatory response syndrome) -criteria [28]. We classified patients having sepsis if they had infection diagnosis and fulfilled at least two SIRS criteria, severe sepsis if they had sepsis and at least one non-cardiovascular organ failure defined by organ specific SOFA score 3 or 4, and septic shock if they had sepsis and the cardiovascular SOFA score was 3 or 4. We only included patients with emergency admission in the final analysis regarding sepsis in order to enhance the aptness of the retrospective sepsis classification.

We performed statistical analysis using SPSS Statistics 19.0 (SPSS Inc., Chicago, IL, USA). We report con-tinuous data as medians with interquartile range (IQR, 25^th ^to 75^th ^percentiles) and categorical data as percentages and count. We calculated 95% confidence intervals (CI) for the main outcome data. We used Mann-Whitney U-test to compare continuous variables and Chi-square test to compare categorical data. We compared repeated measurements (EQ-5D and VAS scores) with the Wilcoxon signed rank test. Reported *P*-values were two-sided and significance was set at the 0.05 level.

We conducted a multiple logistic regression analysis to assess independent risk factors for hospital mortality of patients treated with RRT. We used a backwards elimination approach and a significance level of < 0.05 for entry and > 0.10 for stepwise removal. We entered the following variables: gender, admission type (surgical/medical), time from hospital admission to ICU admission (days), SAPS II score, SOFA score and presence of severe sepsis. In the first model we found SOFA score not to be associated with mortality and, therefore, performed the second model where we replaced SOFA score with organ-specific variables: need for vasoactive drugs, need for mechanical ventilation, and serum creatinine. We tested the goodness of fit with the Hosmer-Lemeshow C-statistics. We report odds ratios with 95% CI.

## Results

**Figure 1:**
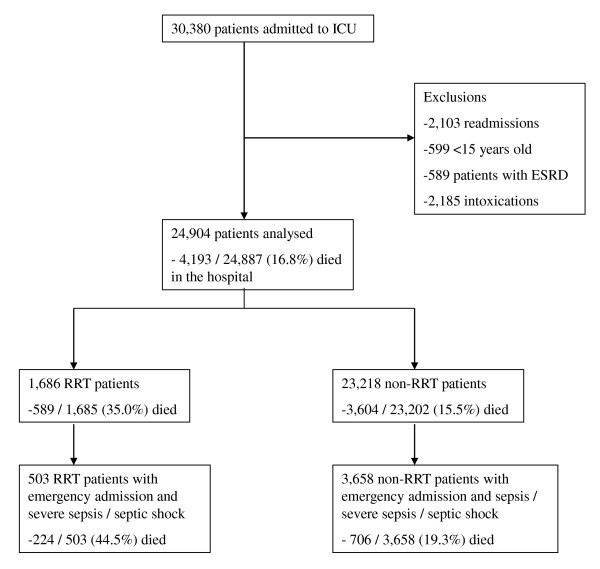
**Flow chart of the patient inclusion and grouping and the hospital mortality in each group**. Mortality is expressed as no./total no (%). ICU, intensive care unit; ESRD, end-stage renal disease; RRT, renal replacement therapy; non-RRT patients, patients who did not need renal replacement therapy.

We analysed the final cohort of 24,904 patients admitted to ICU from 1 January 2007 to 31 December 2008 (Figure 1). Of these patients, 1,686 received RRT due to AKI. The incidence of RRT-treated AKI was 6.8% (95% CI 6.5 to 7.1%) among ≥ 15-year-old general ICU patients, which corresponds to a yearly population-based incidence of 19.2 per 100,000 (95% CI 17.9 to 20.5) among ≥ 15 year old inhabitants in Finland.

Characteristics of the patients are presented in Table 1. No significant difference in the age between patients treated with RRT (RRT patients) and patients who did not require RRT (non-RRT patients) existed (*P *= 0.173). The APACHE III diagnosis groups on admission are presented in Table 2. We were able to classify 85.3% of the cohort according to RIFLE GFR criteria. Patients according to the maximum RIFLE class are presented in Table 3. Of the whole cohort, 5,662 of 21,251 patients (26.6%; 95% CI 26.0 to 27.2%) had AKI according to RIFLE (R-F), and of these AKI patients, 1,120 (19.8%) received RRT. Of all patients with AKI, 66.3%, 79.2% and 90.1% reached their maximum RIFLE-class during the first two, three and five days of ICU treatment, respectively. We identified 1,752 patients with RIFLE-F of whom 776 (44.3%) received RRT. RRT was initiated within the first day in the ICU in 66.4% of the RRT patients. By days 2, 3 and 5 RRT was initiated for 83.3%, 90.1% and 95.1% of the RRT patients.

**Table 1:** Characteristics of patients according to treatment with renal replacement therapy (RRT)

Characteristic	RRT *N *= 1,686	Non-RRT *N *= 23,218	*P*-value
Age - median (IQR) (yr)	63 (52 to 72)	62 (50 to 73)	0.173
Male gender - no./total no. (%)	1,143/1,685 (67.8%)	14,641/23,200 (63.1%)	< 0.001
SAPS II score - median (IQR)	48 (37 to 62)	33 (23 to 46)	< 0.001
SOFA (1.d) score - median (IQR)	10 (7 to 13)	6 (3 to 8)	< 0.001
Mean daily TISS score - median (IQR)	36.4 (29.7 to 43.2)	28.7 (22.4 to 35.0)	< 0.001
Emergency admission - no./total no. (%)	1,558/1,684 (92.5%)	19,122/23,202 (82.4%)	< 0.001
Surgical admission- no./total no. (%)	410/1,685 (24.3%)	9,426/23,208 (40.6%)	< 0.001
Sepsis/severe sepsis - no./total no.(%)	510/1,681 (30.3%)	3,753/23,101 (16.2%)	< 0.001
Mechanical ventilation - no./total no. (%)	1,014/1,642 (61.8%)	11,116/22,409 (49.6%)	< 0.001
Vasoactives-no./total no. (%)	1,067/1,671 (63.9%)	9,556/23,175 (41.2%)	< 0.001
Creatinine - median (IQR) (micromol/L)	210 (119 to 352)*	72 (56 to 101)†	< 0.001
Urine output - median (IQR) (mL/d)	829 (232 to 1,947)	2,355 (1,555 to 3,390)	< 0.001

IQR, Interquartile range (25^th ^to 75^th ^percentiles); SAPS, Simplified Acute Physiology Score; SOFA, Sequential Organ Failure Assessment; TISS, Therapeutic Intervention Scoring System. * Data missing for 5% of cases. †Data missing for 7% of cases.

**Table 2:** The APACHE (Acute Physiology and Chronic Health Evaluation) III diagnostic groups of the patients

	RRT	Non-RRT
Total no. of patients*	1,685	23,208
Non-operative	1,275 (75.7%)	13,782 (59.4%)
Cardiovascular	153 (9.1%)	3,111 (13.4%)
Gastrointestinal	149 (8.8%)	1,338 (5.8%)
Hematological	22 (1.3%)	65 (0.3%)
Metabolic	102 (6.1%)	891 (3.8%)
Neurological	74 (4.4%)	2,423 (10.4%)
Renal	257 (15.3%)	159 (0.7%)
Respiratory	146 (8.7%)	2,572 (11.1%)
Sepsis	284 (16.9%)	1,110 (4.8%)
Trauma	25 (1.5%)	1,115 (4.8%)
Other	44 (2.6%)	661(2.8%)
Missing	19 (1.1%)	337 (1.5%)
Postoperative	410 (24.3%)	9,426 (40.6%)
Cardiovascular	177 (10.5%)	2,947 (12.7%)
Gastrointestinal	137 (8.1%)	2,290 (9.9%)
Gynecological	8 (0.5%)	313 (1.3%)
Neurological	29 (1.7%)	2,060 (8.9%)
Orthopedic	7 (0.4%)	391 (1.7%)
Renal	12 (0.7%)	224 (1.0%)
Respiratory	13 (0.8%)	563 (2.4%)
Trauma	11 (0.7%)	419 (1.8%)
Missing	16 (0.9%)	219 (0.9%)

RRT; renal replacement therapy, non-RRT; patients without renal replacement therapy * Data on APACHE III diagnosis group were missing for one RRT patient and 10 non-RRT patients.

**Table 3:** Patients classified according to RIFLE (Risk, Injury, Failure) glomerular filtration rate criteria

	All (*N *= 24,904)	RRT (*N *= 1,686)	RRT, before*	Non-RRT (*N *= 23,218)
No AKI	15,589 (73.4%)	219 (16.4%)	147 (19.8%)	15,370 (77.2%)
Risk	2,198 (10.3%)	100 (7.5%)	62 (8.4%)	2,098 (10.5%)
Injury	1,712 (8.1%)	244 (18.2%)	150 (20.2%)	1,468 (7.4%)
Failure	1,752 (8.2%)	776 (58.0%)	383 (51.6%)	976 (4.9%)
No data	3,653 (14.7%)	347 (20.6%)	944 (56%)	3,306 (14.2%)

AKI, Acute kidney injury; RRT; renal replacement therapy, non-RRT; patients without renal replacement therapy *RIFLE class before the initiation of RRT

Outcome data and data on HRQoL as measured with the EQ-5D index and VAS scores are presented in Table 4, and the responses of the EQ-5D questionnaire at six months in Additional file 1. Hospital mortality of RRT patients stratified according to the timing of RRT initiation was 33.1% in patients with early (days 1 to 2) RRT, 39.8% with delayed (days 3 to 5) RRT, and 53.5% with late (from day 6 onwards) RRT (*P *< 0.001).

**Table 4:** Outcome of patients according to treatment with renal replacement therapy (RRT)

	RRT *N *= 1,686	Non-RRT *N *= 23,218	*P*-value
ICU stay - median (IQR) days	5.2 (1.9 to 10.8)	1.5 (0.9 to 3.2)	< 0.001
Hospital stay - median (IQR) days	16 (8 to 29)	9 (5 to 16)	< 0.001
Treatment restricted -no./total no. (%)	388/1,533 (25.3%)	2,665/21,339 (12.5%)	< 0.001
Hospital mortality -no./total no. (% (95%CI))	589/1,685 (35.0% (32.7 to 37.3%))	3,604/23,202 (15.5% (15.0 to 16.0%))	< 0.001
Hospital mortality -No AKI according to RIFLE -no./total no. (%)	40/219 (18.3%)	1,573/15,364 (10.2%)	< 0.001
Hospital mortality -RIFLE -Risk -no./total no. (%)	40/100 (40%)	480/2,095 (22.9%)	< 0.001
Hospital mortality -RIFLE -Injury -no./total no. (%)	128/244 (52.5%)	430/1,468 (29.3%)	< 0.001
Hospital mortality -RIFLE -Failure -no./total no. (%)	245/776 (31.6%)	326/976 (33.4%)	0.417
SAPS II based SMR - (95% CI)	0.76 (0.70 to 0.82)	0.61 (0.59 to 0.63)	
Six-month mortality -no./total no. (% (95%CI))	699/1,415 (49.4% (46.8 to 52.0%))	5,101/18,367 (27.8% (27.2 to 28.5%))	< 0.001
EQ-5D index at baseline - median (IQR) - no./total no. (%)	0.68 (0.49 to 1.0) 431/716 (60.2%)	0.69 (0.53 to 1.0) 7,487/13,266 (56.4%)	0.004
EQ-5D index at six months - median (IQR) - no./total no. (%)	0.63 (0.49 to 0.79) 313/716 (43.7%)	0.68 (0.52 to 1.0) 5,415/13,266 (40.8%)	0.015
VAS at baseline - median (IQR) - no./total no. (%)	60 (40 to 80) 223/716 (31.1%)	70 (50 to 80) 4,505/13,266 (34.0%)	0.009
VAS at six months - median (IQR) - no./total no. (%)	70 (50 to 80) 274/716 (38.3%)	70 (55 to 85) 4,841/13,266 (36.5%)	0.059

Quality of life is measured with the EuroQol (EQ-5D) index and with the visual analogue scale (VAS) score. AKI; Acute kidney injury, IQR; interquartile range (25th to 75th percentiles), RIFLE, Risk, Injury, Failure -classification; SAPS, Simplified Acute Physiology Score; SMR, standardised mortality ratio. Treatment restrictions include: 1) no resuscitation, 2) no increase in the intensity of treatment, 3) treatment withdrawal.

In multiple logistic regression analysis the time from hospital admission to ICU admission (days) and SAPS II score on admission (one point increments) were independently associated with increased risk for hospital mortality of RRT patients. The odds ratios (95% CI) were 1.055 (1.029 to 1.081) (*P *< 0.001) per one day and 1.056 (1.048 to 1.065) (*P *< 0.001) per one SAPS II point, respectively. Higher serum creatinine and absence of severe sepsis were associated with a decreased risk for hospital death with odds ratios (95% CI) of 0.998 (0.997 to 0.999) per each micromol/l (*P *< 0.001) and 0.682 (0.528 to 0.881) (*P *= 0.003), respectively. The Hosmer-Lemeshow C-statistics for the final model was 5.771 (*P *= 0.673).

Of the 1,553 RRT patients with emergency admission, 503 (32.4%) had severe sepsis. Characteristics and outcome data of RRT patients with and without severe sepsis are presented in Additional file 2. Hospital mortality in RRT patients with severe sepsis was 44.5% (40.2 to 48.8%), and in non-RRT patients with sepsis or severe sepsis 19.3% (18.0 to 20.6%) (*P *< 0.001).

## Discussion

This large, retrospective multi-centre cohort study included all Finnish adult general ICU patients in a period of two years. We found that the population-based incidence of AKI treated with RRT was 19.2 per 100,000 among ≥ 15-year-old inhabitants in Finland. During the study period, 6.8% of patients admitted to general ICUs were treated with RRT and 26.6% had AKI according to RIFLE classification. Hospital mortality of RRT patients was significantly higher compared to non-RRT patients, 35.0% vs. 15.5%.

The previously reported population-based incidence of RRT-treated AKI varies from 8 to 30 per 100,000 [5-12]. In the nationwide prospective Australian study by Silvester *et al. *[5] the incidence of AKI treated with RRT in an ICU setting was 8 per 100,000. Furthermore, RRT incidence in the United States in a study based on diagnosis and procedure codes in the Nationwide Inpatient Sample registry was 27 per 100,000 [11]. The study also included patients with chronic kidney disease (CKD) [11]. Incidence of RRT-treated AKI was 28.6 per 100,000 in a prospective Scottish study conducted both in ICU and dialysis unit settings [10]. The population-based incidence of RRT in our study was 19.2 per 100,000, in broad agreement with the previous reports. In Finland, RRT administered due to AKI outside ICUs is rare, and, thus, lack of these data may have caused only a minor bias in the population-based incidence. The proportion of RRT-treated AKI in our study was in line with previous recent reports from other countries [1,3,13,14].

The incidence of AKI defined by RIFLE has been reported to vary between 11 and 67% among ICU patients [2,3,29]. In the study by Joannidis *et al. *[29], the incidence of AKI defined by the GFR criteria alone was lower, 25% compared to 35%, when urine output criteria also were considered. Since we were able to classify patients only on the basis of RIFLE GFR criteria, the proportion of AKI patients may be slightly underestimated, and may explain along with the early initiation of RRT why 16% of the RRT patients did not fill the RIFLE GFR criteria. Interestingly, the mortality of RIFLE-F patients with or without RRT did not differ, and RRT patients with RIFLE-I or RIFLE-R had higher mortality rates than RIFLE-F RRT patients. With urine output criteria these RIFLE-R and RIFLE-I patients might have reached RIFLE-F class. Previously, it has been reported that 14 to 30% of RIFLE-F patients had received RRT [2,3]. In our study, the proportion was greater, although the overall population-based incidence of RRT corresponded with previous studies.

In this study, the hospital mortality of RRT patients with corresponding characteristics and disease severity (SAPS II has ranged from 45 to 48 in studies with reported values [13,17,18]) was lower than in previous studies with mortality rates between 44 and 64% [13,15-19]. The lowest hospital mortality rates have been from 44 [15] to 47% [5]. In both studies half of the patients had severe sepsis [5,15]. Silvester *et al. *[5] reported a mean SAPS II score of 55, which equals the score of the RRT patients with severe sepsis in our study, whose hospital mortality was 44.5%. Waikar *et al. *[11] reported hospital mortality of RRT-treated patients to be only 28.1%, but the study included also patients with CKD. A third of RRT patients in our study had severe sepsis compared to half in previous studies with higher hospital mortality rates [16-18]. A smaller proportion of patients with severe sepsis may have contributed to the lower mortality rate in our study. However, RRT patients with severe sepsis had a lower mortality rate compared to previous reports [16-18]. Hospital mortality of septic AKI patients (of whom 70% received RRT) with corresponding severity of disease has been reported to be 70% [30]. In our cohort, RRT was initiated earlier compared to the study by Bagshaw *et al. *[31]. Tendency to initiate RRT early in Finland offers one possible explanation for better survival. Delannoy *et al. *[21] reported the six-month mortality of RRT-treated AKI patients with median SAPS II score of 63 to be 62%. In our study, the most severely ill patients (RRT patients with severe sepsis) had slightly lower SAPS II scores and a six-month mortality rate of 59%.

The SAPS II based SMR of RRT patients was 0.76 in our study. Previously, Uchino *et al. *[17] have reported a SMR of 1.38 among patients receiving continuous RRT. Silvester *et al. *[5] reported a SAPS II predicted mortality of 51.2% with an observed mortality of 46.8% and in the study by Metnitz *et al. *[13] the SAPS II predicted and observed mortality were 44.7% and 62.8%, respectively. The SMRs yielded from both of these studies remain higher than in our study. It has been discussed that SAPS II may underestimate the mortality of patients with AKI [1], while our results imply that SAPS II generally overestimates the mortality of both RRT and non-RRT patients. Given the lower SMR of RRT patients compared to non-RRT patients, the treatment of RRT patients may not have improved as much as the treatment of non-RRT patients since the validation of SAPS II scoring system.

As we found in the multiple regression analysis, longer time from hospital admission to ICU among patients receiving continuous RRT [17] and high SAPS II score among AKI patients have been associated with increased mortality, while the absence of severe sepsis has been associated with better survival [1]. We found an association with higher creati-nine on ICU admission and decreased risk for mortality, potentially reflecting that patients with higher creatinine had more often isolated AKI. Higher creatinine on the day of RRT initiation has been associated with decreased mortality [31,32], which may be explained by better nutritional status reflecting better overall health status [31,32] or underlying CKD and an acute-on-chronic kidney injury with different prognosis [31]. Lower creatinine and worse outcome may account for volume overload known to be independently associated with increased mortality [31,32].

We were able to obtain the six-month follow-up EQ-5D index score only from 44% of RRT patients, which may have caused bias. Our cohort of 313 patients, however, is the second largest to our knowledge of RRT-treated patients with HRQoL data. In addition, we compared the HRQoL of RRT patients to non-RRT patients. Among cancer patients, 0.06 to 0.08 has been considered as a minimally important clinical difference in the EQ-5D index score and 7 for the VAS score [33]. Several previous studies have reported lower HRQoL among RRT patients compared to matched general population [20,21]. Morsch *et al. *[34] found that younger and less severely ill RRT-treated patients had better HRQoL as measured with the SF-36 instrument than older patients with long hospital stay. Johansen *et al. *[35] reported an extremely low HRQoL among 60-day survivors after RRT. We found no clinically significant difference in EQ-5D score between RRT and non-RRT patients after six months follow-up. Regarding the five dimensions of the EQ-5D index, the groups did not differ in suffering from pain or anxiety, and differences in scores for mobility, self-care and usual activities were small. In addition, the VAS score reflecting the patients own perception of health at six months of RRT patients corresponded with the score of non-RRT patients and the earlier reported score of general population [20].

There are some limitations to our study. First, the data were prospectively collected as a routine set of all ICU admissions and, therefore, not addressed to answer specific questions regarding the received RRT. Accordingly, we lack information on the modality and dose of RRT. Second, we were unable to apply the urine output criteria of RIFLE classifica-tion and obtain a true baseline creatinine. Third, the six-month mortality rate was available only from 80% of the patients. However, our cohort is to the best of our knowledge the largest population reporting six-month mortality data of RRT-treated AKI among critically ill patients.

## Conclusions

In conclusion, the population-based incidence of AKI treated with RRT was 19.2 per 100,000. Of all ICU patients, 6.8% received RRT and 26.6% had AKI according to RIFLE classification. In this unselected population of general ICU patients, the hospital and six-month mortality rates were lower than previously reported for ICU-treated RRT patients.

## Key messages

• Population-based incidence of RRT-treated AKI was 19.2 per 100,000 in Finland and 6.8% of all adult general ICU-patients.

• Hospital and six-month mortality of patients treated with RRT were 35.0% and 49.4%, respec-tively.

• Patients treated with RRT perceived their health related quality of life after six-months follow-up as good as patients treated without RRT and general population.

## Abbreviations

ADQI: Acute Dialysis Quality Initiative; AKI: acute kidney injury; APACHE: Acute Physiology and Chronic Health Evaluation; CKD: chronic kidney disease; ESRD: end-stage renal disease; EQ-5D index: EuroQol-instrument for analysing health related quality of life; GFR: glomerular filtration rate; HRQoL: health related quality of life; ICD-10: International Classification of Diseases, 10^th ^revision; LOS: length of stay; MDRD: Modification in Diet in Renal Disease; non-RRT patients: patients who were not treated with renal replacement therapy; RRT: renal replacement therapy; RIFLE: Risk, Injury, Failure -classification; SAPS: Simplified Acute Physiology Score; SOFA: Sequential Organ Failure Assessment; TISS: Therapeutic Intervention Scoring System; SMR: standardised mortality ratio; VAS: visual analogue scale

## Competing interests

The authors declare that they have no competing interests.

## Authors' contributions

STV carried out the data analysis and drafted the manuscript. VP participated in designing the study and critically revising the manuscript. MR participated in the data analysis and critically revised the manuscript. KMK conceived the study, participated in its design and helped to draft the manuscript. All authors read and approved the final manuscript.

## Supplementary Material

Additional file 1 **EQ-5D health-related quality of life in critically ill patients with renal replacement therapy (RRT) at six months**. Click here for file

Additional file 2 **Characteristics and outcome of critically ill emergency patients treated with renal re-placement therapy according to presence or absence of severe sepsis or septic shock**. Click here for file
