# Supplementary material for: Population-based incidence, mortality and quality of life in critically ill patients treated with renal replacement therapy: a nationwide retrospective cohort study in finnish intensive care units
Source: Crit Care. 2012 Jan 20;16(1):R13. doi: 10.1186/cc11158 (PMC3396249; doi:10.1186/cc11158)
Supplement: Additional file 1 — EQ-5D health-related quality of life in critically ill patients with renal replacement therapy (RRT) at six months. [file cc11158-S1.PDF]

Additional File Table 1.EQ-5D health-related quality of life in critically ill patients with renal replacement therapy (RRT) at six months.

| | RRT (N=313) | Non-RRT (N=5415) | *P*-value |
|---|---|---|---|
| **Mobility** | | | |
| I have no problems in walking about | 131 (41.9%) | 2840 (52.4%) | <0.001 |
| I have some problems in walking about | 157 (50.2%) | 2231 (41.2%) | 0.002 |
| I am confined to bed | 25 (8.0%) | 344 (6.4%) | 0.237 |
| **Self-Care** | | | |
| I have no problems with self -care | 211 (67.4%) | 3946 (72.9%) | 0.037 |
| I have some problems washing or dressing myself | 78 (24.9%) | 1108 (20.5%) | 0.062 |
| I am unable to wash or dress myself | 24 (7.7%) | 361 (6.7%) | 0.485 |
| **Usual Activities***(e.g. work, study, housework, family or leisureactivities)* | | | |
| I have no problems with performing my usual activities | 153 (48.9%) | 3026 (55.9%) | 0.016 |
| I have some problems with performing my usual activities | 108 (34.5%) | 1773 (32.7%) | 0.536 |
| I am unable to perform my usual activities | 52 (16.6%) | 616 (11.4%) | 0.008 |
| **Pain/Discomfort** | | | |
| I have no pain or discomfort | 128 (40.9%) | 2351 (43.4%) | 0.412 |
| I have moderate pain or discomfort | 171 (54.6%) | 2744 (50.7%) | 0.181 |
| I have extreme pain or discomfort | 14 (4.5%) | 320 (5.9%) | 0.384 |
| **Anxiety/Depression** | | | |
| I am not anxious or depressed | 214 (68.4%) | 3790 (70.0%) | 0.568 |
| I am moderately anxious or depressed | 88 (28.1%) | 1453 (26.8%) | 0.646 |
| I am extremely anxious or depressed | 11 (3.5%) | 172 (3.2%) | 0.740 |

Brooks R: **EuroQol: the current state of play.** Health Policy 1996, **37**(1):53-72.
