# Supplementary material for: Population-based incidence, mortality and quality of life in critically ill patients treated with renal replacement therapy: a nationwide retrospective cohort study in finnish intensive care units
Source: Crit Care. 2012 Jan 20;16(1):R13. doi: 10.1186/cc11158 (PMC3396249; doi:10.1186/cc11158)
Supplement: Additional file 2 — Characteristics and outcome of critically ill emergency patients treated with renal re-placement therapy according to presence or absence of severe sepsis or septic shock. [file cc11158-S2.PDF]

Additional File Table 2. Characteristics and outcome of critically ill emergency patients treated with renal replacement therapy according to presence or absence of severe sepsis or septic shock.

	Severe sepsis / septic shock N=503	No sepsis N=1050	P-value
Age - median [IQR] (yr)	61 (53-71)	63 (51-72)	0.182
Male gender – no./total no. (%)	342 / 503 (68.0%)	705 / 1049 (67.2%)	0.757
SAPS II score – median [IQR]	55 (44-67)	48 (36-61)	<0.001
SOFA (1.d) score – median [IQR]	12 (9-14)	9 (6-12)	<0.001
Mean daily TISS score – median [IQR]	40.4 (35.6-46.2)	33.6 (26.8-40.6)	<0.001
Surgical admission % – no./total no. (%)	92 / 503 (18.3%)	197 / 1049 (18.8%)	0.817
Mechanical ventilation – no./total no. (%)	388 / 500 (77.6%)	546 / 1014 (53.8%)	<0.001
Vasoactives –no./total no. (%)	403 / 498 (80.9%)	585 / 1043 (56.1%)	<0.001
Creatinine – median [IQR] (micromol/L)	203 (132-314)	230 (124-393)*	0.005
Urine output – median [IQR] (mL/d)	825 (230-1779)	758 (215-1935)	0.940
ICU stay – median [IQR] days	8.0 (4.0-13.1)	4.0 (1.6-9.3)	<0.001
Hospital stay – median [IQR] days	18 (9-31)	15 (8-29)	0.076
Hospital mortality –no./total no. (% [95% CI])	224 / 503 (44.5% [40.2-48.8 %])	328 / 1049 (31.3% [28.5-34.1%])	<0.001
6-month mortality –no./total no. (% [95% CI])	249 / 422 (59.0% [54.3-63.7%])	405/ 877 (46.2% [42.9-49.5%])	<0.001

IQR; interquartile range (25th-75th percentiles), SAPS; Simplified Acute Physiology Score, SOFA; Sequential Organ Failure Assessment, TISS; Therapeutic Intervention Scoring System.

* Data missing for 6% of patients.
